# Prevalence, Antecedents, and Consequences of Workplace Bullying among Nurses—A Summary of Reviews

**DOI:** 10.3390/ijerph19148256

**Published:** 2022-07-06

**Authors:** Hongli Sam Goh, Siti Hosier, Hui Zhang

**Affiliations:** 1 IPE Management School Paris, 21 Rue Erard, 75012 Paris, France; nurghs@gmail.com; 2 Alice Lee Centre for Nursing Studies, Yong Loo Lin School of Medicine, National University of Singapore, Singapore 117597, Singapore; nurzh@nus.edu.sg

**Keywords:** workplace bullying, systematic review, nurses, overview of reviews

## Abstract

Despite over 25 years of extensive research about the workplace bullying phenomenon in various disciplines, there have been mixed conclusions about its prevalence, antecedents, and consequences among nurses reported by multiple systematic reviews. This summary review used the Cochrane’s Overview of Reviews method to examine the prevalence, antecedents, coping behaviors, and consequences of workplace bullying among nurses to understand the interplay of these variables in healthcare workplace contexts. A total of 12 systematic reviews published between 2013 and 2020 were included based on the eligibility criteria. There were differences in workplace bullying prevalence across different reviews, ranging from 1 to 90.4%, but a more recent review estimated the pooled prevalence at 26.3%. This review identified at least five main types of antecedents for workplace bullying: demographics, personality, organizational culture, work characteristics, and leadership and hierarchy. Workplace bullying affected nurses, organizational outcomes, and patient safety. This review proposes an integrative model to explain workplace bullying among nurses and highlights the need for more studies to evaluate interventions to address this phenomenon.

## 1. Introduction

Nursing has long been recognized as a challenging career, which is beset with workplace adversities, such as stress and bullying, of which the latter warrants a cause for concern. Nurse bullying is not new and has been the subject of research studies for over 25 years. This phenomenon was suggested to affect nurses in the United States (US) more than 100 years ago based on a New York Times article in 1909, “The hospital tyrants” [1]. Unfortunately, despite years of research in this area, nurses continue to experience bullying today as many leaders, institutions, and even the nurses themselves either deny its existence or accept it as the norm, creating a culture of silence that impedes solutions to the problem [1].

Within the broader literature, Nielsen and Einarsen defined workplace bullying as extensive exposure to repeated negative behaviors at the workplace, leaving individuals to feel defenseless against such behaviors [2]. Within the nursing literature, workplace bullying is an umbrella term for most types of workplace aggression and violence, ranging from emotional neglect to threats of violence and physical assault [3]. Terms that fall under this umbrella include incivility, harassment, and workplace violence. The subject has been extensively studied internationally, across disciplines, particularly within healthcare settings [3]. Workplace bullying occurs when individuals perceive that they are the target of negative actions from one or more persons over time.

According to Trépanier et al. [4], up to 40% of nurses faced bullying behaviors at work, while Houck and Colbert [3] reported prevalence rates ranging from 26 to 77%. These figures suggest that the healthcare industry seems to be acutely affected by this phenomenon. In contrast, a systematic review, which examined non-healthcare studies, reports an estimated global prevalence of only 15%, suggesting that workplace bullying in general workplace settings might be less prevalent than in a healthcare context [5]. The high bullying prevalence rate reported among nurses warrants an urgent need for nurse leaders to address this issue.

The high prevalence rate of workplace bullying among nurses is alarming given the consequences and impact on nurses and organizations. Exposure to bullying is associated with symptoms of depression, anxiety, and psychological distress in nurses [6,7], as well as somatic physical health problems, including insomnia and headache [8]. Workplace bullying can also undermine nurses’ professional well-being, decreasing engagement and quality of work motivation, and increasing absenteeism, turnover, and burnout symptoms [4,9,10].

There have been multiple systematic reviews that evaluated workplace bullying in nursing. Most of them reported mixed or inconclusive findings of the prevalence, antecedents, and consequences to address workplace bullying due to the heterogeneity in study designs, measurement instruments, and contextual variations across the included studies. For example, some reviews examined workplace bullying prevalence only, while others focused on its triggering factors. Still, other reviews only focus on specific consequences of workplace bullying [1,2]. The different reviews make it difficult for nursing leaders to comprehend the scope and extent of workplace bullying, much less know how to manage or address it. Castronovo et al. [1] lamented the persistent existence of these problems despite years of research in this area. In light of the varied conclusions, we decided to conduct a summary review with the aim of summarizing the findings from existing systematic reviews, which examined the prevalence, antecedents, and consequences of workplace bullying among nurses to understand the interplay of these variables within healthcare. At the end of the review, these findings will be used to develop a theoretical framework for analyzing workplace bullying in nursing.

## 2. Materials and Methods

The summary review of systematic reviews was conducted using Cochrane’s Overview of Reviews method to synthesize reviews examining workplace bullying and its prevalence, trend, antecedents, consequences, and interventions. There have been extensive publications of studies in nursing and healthcare literature. This method was adopted because it provides an explicit and structured approach to extract and analyze results across the topic of interest [11]. As there have been multiple reviews that focus on different aspects of workplace bullying, this method allows us to compare strengths of evidence derived from varied review designs to draw meaningful conclusions. Finally, the Cochrane Overview of Reviews method allows us to summarize the findings from different reviews about workplace bullying for clinicians and decision-makers rather than leaving them to assimilate the results of multiple systematic reviews themselves [12]. The Cochrane’s Overview of Reviews method comprises five steps: (i) defining the review and questions; (ii) outlining the search strategy to retrieve systematic reviews (with or without meta-analyses); (iii) establishing clear eligibility criteria for article selection; (iv) extraction of data from each review, including its characteristics, risk of bias and outcomes; and (v) collation and summary of results in accordance to the specific objectives or questions of the review [11].

### 2.1. Defining the Review Questions

Three questions for the summary review were developed based on the authors’ preliminary literature review:

1. What are the prevalence and trends in workplace bullying among nurses?
2. What are the antecedents for workplace bullying among nurses?
3. What are the consequences of workplace bullying for nurses?

### 2.2. Search Strategy

A comprehensive literature search was conducted between April 2021 and December 2021 to search for relevant systematic reviews using the following key search terms and related text words: ‘workplace bullying,’ ‘nurs*,’ and ‘review.’ The search for literature was limited to those published within the past ten years, as this paper aimed to provide a comprehensive review of all recently published reviews on nurse bullying. A total of seven electronic databases were searched: PubMed, ScienceDirect, Medline, Scopus, CINAHL, Web of Science, and PsycINFO. The search was conducted using different combinations of exact keywords on titles and abstracts. Thereafter, the retrieved articles were screened for relevance to the review questions.

### 2.3. Article Selection

**Figure 1:**
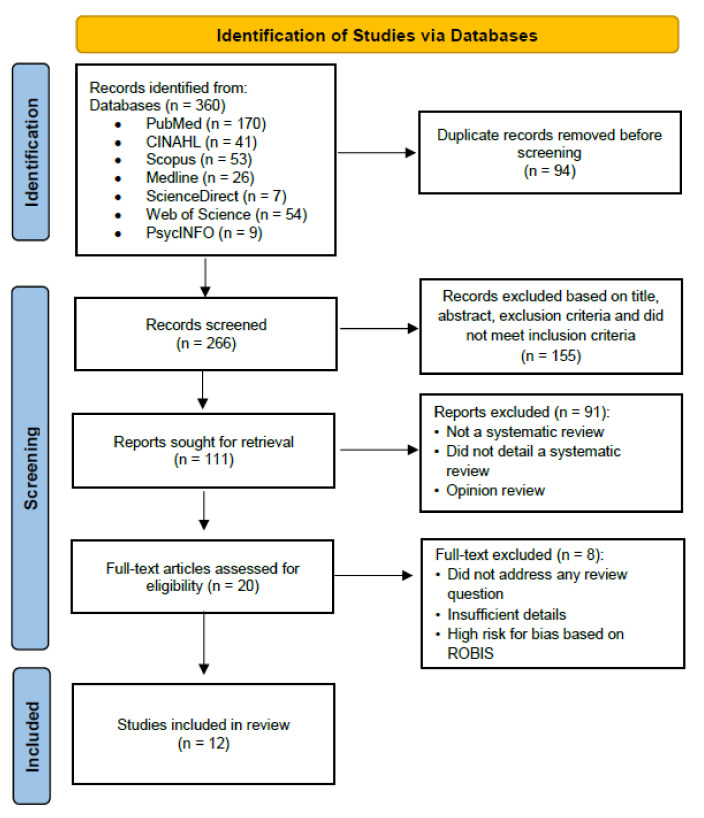
PRISMA diagram.

The selection of the studies was conducted independently by two authors based on the eligibility criteria. Disagreement during the selection was resolved by discussion with a third-party arbiter. The inclusion criteria were: (i) derived from a systematic review; (ii) involved nursing professionals; (iii) addressed the review questions; and (iv) published in English. In addition, we excluded studies that were merely literature reviews or any other review that did not demonstrate a systematic process, did not focus on nurses, or were published in other languages with no English translation. The decision-making process and the search results at each step of the course are depicted in the PRISMA (Preferred Reporting Items for Systematic reviews and Meta-Analyses) diagram (Figure 1).

### 2.4. Data Extraction and Quality Assessment

Data were extracted by one author (SG) and verified by another (ZH) for relevancy and accuracy. The two authors then independently extracted the data, including the review objectives, design, search strategy, number of included studies and sample size, geographical location, main findings, and quality appraisal using the ROBIS tool. The ROBIS tool was developed by clinicians at Bristol Medical School with the aim of providing an effective yet robust method to assess the risk of bias for systematic review, and has been recommended by Cochrane for the review method [13]. The tool can also be used to compare the overall risk of bias across various reviews to derive meaningful comparison and contrast of the various findings [13]. When there was a disagreement on the quality of an article, this would be resolved through discussion with a third author (SH) until consensus was achieved. An annotated bibliography was developed to tabulate the characteristics and findings of the studies. The reference management software Mendeley and Microsoft Excel were used to sort the records.

## 3. Results

A total of 12 reviews about workplace bullying were included in this summary review (Table 1). The types of reviews included were: quantitative systematic reviews (*n* = 2), mixed-methods systematic reviews (*n* = 1), integrative reviews (*n* = 5), narrative reviews or systematic reviews with qualitative synthesis (*n* = 3), and scoping reviews (*n* = 1). The samples ranged from 61 to 151,347 participants, and the number of databases searched among the reviews ranged from 3 to 8. The reviews were published between 2013 and 2020, and included studies that were published from the earliest date to 2019. With the exception of one review, which focused solely on studies from Australia [14], most reviews included studies from different countries. Most of the studies were conducted in North America, Europe, and Australia compared to other regions. Rutherford et al. attributed this observation to the inclusion criteria of mostly English-language papers by most reviews and that most journals and databases use English for communication [15]. The summary review also assessed the methodological quality of all 12 reviews, as shown in Table 2. Based on the overall quality assessment of the included reviews, only one review was at low risk of bias for the overall study [16].

### 3.1. Question 1—What Are the Prevalence and Trends in Workplace Bullying among Nurses?

Seven reviews addressed the prevalence of workplace bullying within the nursing and healthcare literature (Table 3). Two reviews conducted a pooled estimation of workplace bullying prevalence and reported a mean prevalence of 26.3 and 66.9% among nurses [8,18]. Spector et al. seemed to be the most comprehensive review between these two reviews, having conducted a quantitative review of 136 healthcare studies on the global nursing violence literature to examine the extent (prevalence), sources, and subtypes of bullying and violence across countries and prevalence. They reported workplace bullying prevalence ranges from 57.6% in hospital settings to 67.7% in psychiatric settings. The mean percentage of perceived bullying also varied across different geographical regions: Middle East (86.5%), Anglo (39.5%), Asia (29.8%), and Europe (8.8%). The highest rate of non-physical violence from peers and colleagues occurred among nurses working in Asia (50.2%), followed by the Middle East (44.9%), Anglo countries (US, Canada, UK, and Australia) (37.4%), and Europe (27.6%). Asian, Anglo, and Middle Eastern nurses suffered similar rates of physical violence at 7.3, 6.6, and 6.0%, respectively. Similarly, a more recent quantitative systematic review involving 45 studies reported a lower percentage of workplace bullying among nurses. They have classified workplace bullying in general terms, demonstrating that the trend in workplace prevalence among nurses has remained vastly varied across different regions [8].

There were vast differences in workplace bullying prevalence across all seven reviews, with one review reporting the greatest prevalence range from 1 to 90.4% [8]. Other reviews also reported a similar prevalence range [8,22]. The vast discrepancies in the reported bullying rates across different nursing studies might suggest regional and country differences in the workplace bullying incidence rates and sources of violence, making it difficult for researchers to grasp its extent and impact. One possible explanation for such discrepancies could be that some countries or cultures may trivialize or pay little attention to the problem, leading to under-reporting issues (Spector et al., 2014). Another reason could be the different ways bullying is defined and measured, inconsistent research methods, and an absence of longitudinal studies [24]. The current lack of local data on the extent of the phenomenon could impede nursing leaders from developing and implementing tailored interventions to address these issues in their specific settings.

Workplace bullying seems more prevalent in hospitals’ high-stress work environments, such as emergency departments, operating theaters, intensive care units, and surgical and psychiatric settings [20,22,23]. However, this trend might not be generalizable across different countries, as Bambi et al. highlighted obstetrics wards as the most affected units in public hospitals in Cape Town, South Africa. Additionally, it appears that nurses in Asian and Middle Eastern countries have a higher prevalence of workplace bullying, and physical and non-physical violence than their counterparts from other regions [8,18].

**Table 3:** Summary table of prevalence rate for workplace bullying among nurses.

No.	Evidence/Reference	Prevalence Rate
1.	Spector et al. (2014) [18]	Prevalence rate: 25–66.9%. Specific: Physical violence (36.4%), non-physical (66.9%), bullying and others (39.7%), sexual harassment (25%), injured (32.7%).
2.	Houck and Colbert (2017) [3]	Prevalence of bullying among nurses was observed to be between 26% and 77%.
3.	Bambi et al. (2018) [20]	% of bullying prevalence: 2.4 to 81%. % of workplace incivility: 67.5 to 90.4%. % of lateral violence (peer violence): 1 to 87.4%.
4	Hartin et al. (2018) [14]	61% of respondents in Australia reported workplace bullying events within the last 12 months.
5.	Hawkins et al. (2019) [22]	Prevalence ranged widely from 0.3 to 73.1% (variations attributed to the workplace context and instrument measuring workplace bullying events [e.g., daily basis, over the past 1 month, or over the past 12 months]). Studies measuring workplace bullying within past 6–12 months reported a more consistent prevalence ranging from 25.6 to 73.1%.
6.	Lever et al. (2019) [8]	Bullying prevalence ranged from 3.9 to 86.5%, with a pooled mean estimate of 26.3%. The pooled mean prevalence of bullying by region: Asia (47.1%), Australia (36.1%), Europe (18.4%), and North America (24.5%).
7.	Johnson and Benham-Hutchins (2020) [23]	% of bullying prevalence in emergency department setting: 60%. % of bullying prevalence in Operating Room setting: 59% witnessed workplace bullying events, but only 6% self-reported such events in the USA.

### 3.2. Question 2—What Are the Antecedents for Workplace Bullying among Nurses?

Five reviews identified five antecedents for workplace bullying within the nursing and healthcare literature (Table 4). Among the five reviews, the most comprehensive was Karatuna et al.’s scoping review, which included 166 studies on workplace bullying among nurses. The review was also the most recent, with included studies published between 2001 and 2019. Hence, we used their review to guide the categorization of antecedents into five main types: demographics, personality, organizational culture, work characteristics, and leadership. These five antecedents can also be grouped under two main layers of antecedents—individual-level or organizational-level [21].

#### 3.2.1. Individual-Level Antecedents

Individual antecedents include demographics and personality traits of individuals who contributed to the occurrence of workplace bullying. The results showed some similarities in the demographical antecedents of bullying across clusters that differ in their cultural practices. In terms of demographics, they found that most studies reported no associations between gender, education level, marital status, and workplace bullying. Conversely, age and length of experience/service were found to be negatively associated with workplace bullying. Other demographical antecedents were found to vary across different geographical clusters and subject to the different socio-cultural and politico-economic influences. For example, nurses considered “vulnerable” to workplace bullying in Anglo countries belong to a certain race, ethnicity, or disability, while those in Latin America and Eastern Europe have children. For personality characteristics, nurses with less locus of control, psychological capital, or poor compliance to social norms were associated with a greater risk of workplace bullying than others [16].

#### 3.2.2. Organizational-Level Antecedents

Organizational-level antecedents included leadership, work characteristics, and organizational culture. For example, an organizational culture that is performance-oriented is more likely to tolerate workplace bullying, while cultures that emphasize people-orientation tolerate such behaviors if the group views the victim as inconsistent with social norms or misaligned with the organizational structure and hierarchy [16]. These findings highlighted group inclusivity within the organization, which is highly dependent and varies according to the larger socio-cultural context.

As for work characteristics, Karatuna et al. (2020) reported that negative work environments and characteristics include work overload, staffing shortages, and stressful working conditions. These variables were found to be reported across all clusters. Trépanier et al. [4] conducted a systematic literature review specifically examining work-related antecedents of workplace bullying in nursing and retrieved 12 relevant studies. They reported similar results to Karatuna et al. based on their four categories of work-related antecedents: (1) job characteristics, (2) quality of interpersonal relationships, (3) leadership styles, and (4) organizational culture. They found that nurses’ better job characteristics, higher quality of interpersonal working relationships, people-centric leadership styles, and positive organizational culture (promoting staff empowerment, distributive justice, and zero tolerance for bullying) were associated with less workplace bullying. Pfeifer and Vessey [19] conducted an integrative review focusing on examining bullying issues among nurses in Magnet^®^ organizations, which are designated hospitals that meet the quality benchmark for providing quality of care and nursing excellence. They found 11 articles (eight quantitative and three qualitative studies). Their review demonstrated emerging evidence on how a positive work environment could contribute to reduced reports of verbal abuse, incivilities, and hostile encounters from colleagues. Despite the positive and significant findings, Pfeifer and Vessey cautioned that workplace bullying can still affect nurses in the Magnet^®^ environment and highlighted the complex interplay of individual and organizational factors in influencing the occurrences of workplace bullying [19].

Leadership and hierarchy seem to mediate in organizational culture and work characteristics. For example, Karatuna et al. reported that autocratic, unsupportive, and disengaged leadership perpetuates high-power distance clusters and increased bullying behaviors [16]. On the other hand, Trépanier et al. [4] found three studies examining how authentic (positive) leadership significantly reduced workplace bullying and burnout reports. All four reviews stated positive leadership mediated the workplace environmental factors by promoting a climate of trust, positive collegial relationships, and mitigating stressful work environments and workplace bullying events [4,16,21,22].

**Table 4:** Summary table of antecedents for workplace bullying.

No.	Types of Antecedents	Subtypes	Association	Evidence
1.	Demographics (Individual-level)	Age	Negatively associated with workplace bullying.	Karatuna et al. (2020) [16] Crawford et al. (2019) [21]
		Length of experience/service	Negatively associated with workplace bullying.	Karatuna et al. (2020) [16]
		Gender	No association.	Karatuna et al. (2020) [16]
		Marital status	No association.	Karatuna et al. (2020) [16]
		Education level	No association.	Karatuna et al. (2020) [16]
		Minority race or ethnicity	Association reported in Anglo, Southern Asia.	Karatuna et al. (2020) [16]
		Disability	Association reported in Anglo.	Karatuna et al. (2020) [16]
		Having children	Association reported in Latin America and Eastern Europe.	Karatuna et al. (2020) [16]
2.	Personality (Individual-level)	Locus of control/assertiveness	Lower locus of control (assertiveness) is negatively associated with workplace bullying.	Karatuna et al. (2020) [16]
		Psychological capital	Less psychological capital is negatively associated with workplace bullying.	Karatuna et al. (2020) [16]
		Vulnerable traits or personality/poor compliance to social norms	Negatively associated with workplace bullying.	Karatuna et al. (2020) [16]
3.	Organizational culture (Organizational-level)	Organizational culture promotes staff empowerment, distributive justice, and zero tolerance for bullying/Magnet^®^ organizational culture	Perceived healthy work environment is negatively associated with workplace bullying.	Karatuna et al. (2020) [16] Pfeifer and Vessey (2017) [19]
		Quality of interpersonal relationships	Association varies according to regions. Vertical bullying was most prevalent in higher power distance cultures, whereas horizontal bullying was either more or equally prevalent in lower power distance cultures.	Crawford et al. (2019) [21] Hawkins et al. (2019) [22]
4.	Work characteristics (Organizational-level)	Work overload	Higher workload is positively associated with workplace bullying.	Karatuna et al. (2020) [16] Trépanier et al. (2016) [4]
		Staff shortages	More severe staff shortages are positively associated with workplace bullying.	Trépanier et al. (2016) [4]
		Stressful working conditions	High-stress work environment is positively associated with workplace bullying.	Trépanier et al. (2016) [4]
5.	Leadership and hierarchy (Organizational-level)	Leadership styles	Autocratic, unsupportive, and disengaged leadership tends to perpetuate high-power distance clusters and increased bullying behaviors.	Trépanier et al. (2016) [4] Crawford et al. (2019) [21] Hawkins et al. (2019) [22] Karatuna et al. (2020) [16]

### 3.3. Question 3—What Are the Consequences of Workplace Bullying for Nurses?

The workplace culture and pervasive nature of bullying have a significant negative impact on nurses, organizations, and patient outcomes. Nine reviews reported the consequences of workplace bullying among nurses [3,8,14,16,17,20,21,22,23]. The summary review generated five types of consequences: psychosocial well-being, physical well-being, work performance, organizational impact, and patient outcomes (Table 5).

#### 3.3.1. Psychosocial Well-Being

From the literature, workplace bullying affects nurses’ psychosocial well-being. Hartin et al. [25] conducted an integrative review of 23 Australian nursing studies. They reported that nurses who experienced workplace bullying faced greater risks of poor psychosocial outcomes such as psychological distress, depression, and burnout. It also undermines the nurses’ professional confidence and decreases their self-worth, motivation, and work ethic. In another systematic review, Johnson and Benham-Hutchins [23] reported similar psychosocial consequences of bullying, including increased stress, somatic symptoms, frustration, absenteeism, and lack of concentration. These findings were retrieved from 14 relevant nursing studies conducted in multiple healthcare settings, suggesting the significance of the issues in nursing. Of the nursing population, Hawkins et al. [22] suggested that workplace bullying might affect new graduate nurses, particularly as this group mainly holds subordinate positions and experiences much uncertainty during their adaption to the workplace. They conducted an integrative review of studies that examined this phenomenon among new graduate nurses and found 16 studies from Canada, the US, Australia, Korea, Singapore, and Ireland. They reported similar consequences on the new nurses, specifically, job satisfaction, burnout, intention to leave, and turnover.

#### 3.3.2. Physical Well-Being

Based on two reviews, workplace bullying is also reported to affect nurses’ physical well-being. The review by Johnson and Benham-Hutchins [23] found one study that surveyed 248 nurses in the Midwest US using an electronic questionnaire and found that work-related bullying showed a highly significant positive relationship with psychological/behavioral responses. However, they did not specify the types of physical outcomes being affected. In another review, Karatuna et al. [16] reported headache, tachycardia, fatigue, sleep disorders, and pseudo-neurological and gastrointestinal complaints as common physiological health outcomes of workplace bullying in their review of 166 studies in different countries. Lever et al. conducted a systematic review specifically looking at the health consequences in the healthcare workplace [8]. They retrieved 45 studies published between 2005 and 2017, with 40 studies examining mental health outcomes and 15 on physical health. They reported that nurses who encountered workplace bullying face a greater risk of developing sleep-related issues, headaches, gastrointestinal problems, and to a lesser extent, back and joint pain and blood pressure changes. As a result, these staff are more likely to report sick leave than those not affected by workplace bullying [8].

#### 3.3.3. Work Performance

The review outlines two types of organizational-related consequences from the review. The first is about the nurses’ work performance. Workplace bullying reduces nursing performance by affecting nurses’ state of mind and impairs their ability to seek help at work, engage in effective and timely communication, and make clinical judgments. As a result, nurses cannot deliver patient care in a safe and effective manner. Hutchinson and Jackson [17] conducted a mixed-methods systematic review to determine how workplace bullying can affect patient care. They found 30 appropriate studies and conducted a content analysis to generate four themes: (1) physician–nurse relations and patient care, (2) nurse–nurse bullying, intimidation, and patient care, (3) reduced nurse performance related to exposure to hostile clinician behaviors, and (4) nurses and physicians directly implicating patients. The first two themes highlighted that physicians and nursing colleagues were the two main sources of bullying behaviors. In comparison, the last two themes revealed how bullying behaviors reduce nurses’ work performance. They reported that nurses affected by workplace bullying were reported to (1) avoid or delay effective communication, (2) experience poor concentration at work, preventing them from delivering safe and effective nursing care, (3) fail to raise safety concerns and seek assistance, and (4) become hostile and perpetrators of similar bullying behaviors.

#### 3.3.4. Organizational Impact

The second organizational-related consequence is the organizational impact. Hartin et al. reported that workplace bullying decreases nurses’ job satisfaction and productivity, such as increased absenteeism and committing errors during work [25]. Johnson and Benham-Hutchins [23] reported that workplace bullying created a negative and hostile work environment, where teamwork and communication are being impeded. Both reviews reported that this indirectly leads to decreased job satisfaction, increased intention to quit, and staff turnover/attrition rate, leading to a higher organizational cost due to recruitment and retention difficulties. Crawford et al. analyzed 21 studies involving nursing students, new graduates, and experienced and academic faculty [21]. They reported that new graduate nurses face a higher risk of workplace bullying and difficulty coping with their new role. This situation is especially significant if the workplace environment is perceived as hostile, toxic, and unforgiving. If not managed properly, these events could negatively impact new nurses’ transition experiences and result in impaired peer relations and even higher staff attrition.

#### 3.3.5. Patient Outcomes

In terms of patient outcomes, workplace bullying indirectly influences patient outcomes by negatively affecting nurses’ work performance. Houck and Colbert conducted an integrative review to examine the association between workplace bullying and patient safety outcomes [3]. They retrieved 11 studies conducted between 1995 and March 2016 in Anglo countries (US, Canada, UK, and Australia). They reported seven patient safety consequences of workplace bullying: (1) patient falls, (2) errors in treatments or medications, (3) patient satisfaction or patient complaints, (4) adverse event or patient mortality, (5) altered thinking or concentration, (6) silence or inhibited communication, and (7) delayed care. Among these themes, the first four were reported as patient-related consequences of workplace bullying. The last three revolved around the negative impact on nursing performance related to patient safety. These findings concur with the review by Hutchinson and Jackson [17] about patient-related consequences. They also reported similar outcomes such as medication errors, surgical errors, and failure to report clinical issues of concern resulting in adverse events. Additionally, Hutchinson and Jackson highlighted how open displays of workplace bullying could erode patients’ confidence in nurses’ capability and instances of how bullied nurses may, in turn, display hostile behaviors or non-emphatic care, resulting in poor patient satisfaction [23].

**Table 5:** Summary table of consequences of workplace bullying.

No.	Types of Consequences	Subtypes	Evidence
1.	Psychosocial well-being	Psychological stress	Hartin et al. (2018) [14]; Bambi et al. (2018) [20]; Hawkins et al. (2019) [22]; Crawford et al. (2019) [21]; Johnson and Benham-Hutchins (2020) [23]
		Depression	Hartin et al. (2018) [14]; Bambi et al. (2018) [20]; Hawkins et al. (2019) [22]
		Burnout	Hartin et al. (2018) [14]; Hawkins et al. (2019) [22]
		Professional confidence	Hartin et al. (2018) [14]
		Sense of self-worth	Hartin et al. (2018) [14]
		Work motivation	Hartin et al. (2018) [14]; Johnson and Benham-Hutchins (2020) [23]
2.	Physical well-being	Sleep-related issues	Karatuna et al. (2020) [16]; Lever et al. (2019) [8]
		Headaches	Karatuna et al. (2020) [16]; Lever et al. (2019) [8]
		Gastrointestinal problems, and to a lesser extent,	Karatuna et al. (2020) [16]; Lever et al. (2019) [8]
		Back and joint pain	Lever et al. (2019) [8]
		Cardiac-related symptoms, tachycardia, or blood pressure changes	Karatuna et al. (2020) [16]; Lever et al. (2019) [8]
		Sick leave/absenteeism	Bambi et al. (2018) [20]; Lever et al. (2019) [8]; Hawkins et al. (2019) [22]; Johnson and Benham-Hutchins (2020) [23]
3.	Work performance	Avoidance behavior, delay in effective communication, or impaired peer relations	Hutchinson and Jackson (2013) [17]; Houck and Colbert (2017) [3]; Crawford et al. (2019) [21]; Johnson and Benham-Hutchins (2020) [23]
		Poor concentration at work, preventing them from delivering safe and effective nursing care	Hutchinson and Jackson (2013) [17]; Houck and Colbert (2017) [3]; Bambi et al. (2018) [20]; Hawkins et al. (2019) [22]; Johnson and Benham-Hutchins (2020) [23]
		Fail to raise safety concerns and seek assistance/delayed care	Hutchinson and Jackson (2013) [17]; Houck and Colbert (2017) [3]; Hawkins et al. (2019) [22]
		Become hostile and perpetrators of similar bullying behaviors	Hutchinson and Jackson (2013) [17]
4.	Organizational impact	Job dissatisfaction	Hartin et al. (2018) [14]; Hawkins et al. (2019) [22]; Crawford et al. (2019) [21]; Johnson and Benham-Hutchins (2020) [23]
		Increased intention to quit	Johnson and Benham-Hutchins (2020) [23]
		Increased staff turnover/attrition rate	Bambi et al. (2018) [20]; Johnson and Benham-Hutchins (2020) [23]; Hawkins et al. (2019) [22]
		Higher organizational costs due to recruitment and retention difficulties	Johnson and Benham-Hutchins (2020) [23]
5.	Patient outcomes	Patient falls	Houck and Colbert (2017) [3]
		Errors in treatments or medications	Houck and Colbert (2017) [3]
		Adverse event or patient mortality	Houck and Colbert (2017) [3]
		Patient satisfaction or patient complaints	Houck and Colbert (2017) [3] Hutchinson and Jackson (2013) [17]

## 4. Discussion

Workplace bullying is a complex and dynamic social phenomenon that generates various definitions and concepts, making it hard to unify or standardize. Instead, our summary review compared nursing and non-healthcare literature to provide an overview of the various concepts and terms about workplace bullying, as shown in Table 6 [2,4,22,26,27,28,29,30,31].

### 4.1. Prevalence and Trends of Workplace Bullying among Nurses

The prevalence rate of workplace bullying varies widely. Nevertheless, there is empirical evidence to show the widespread prevalence of workplace bullying in nursing across different countries and healthcare contexts when the data is considered collectively from the included systematic reviews. The review by Lever et al. [8] showed that the pooled workplace bullying prevalence among nurses is estimated at 26.3%, which was similar to the pooled prevalence rate of 22% as reported by a Korean-language systematic review that examined 23 nursing studies [32]. However, it was higher than the prevalence rate of 11 to 18%, as reported by a non-nursing systematic review and meta-analysis that extracted 86 studies from various industry fields [5]. The higher-than-average prevalence rate observed in the healthcare sector could be attributed to several factors, including the highly stressful environment faced by healthcare professionals around the world, availability of reporting systems, and greater staff willingness to recognize and report workplace bullying events [8,18].

A remarkable proportion of nurses in hospital settings have experienced workplace violence, with bullying being the most common. The international variation in workplace bullying prevalence could be due to differences in sample size, type of measurement used, organizational/service setting, and reporting culture [2,8,18]. We attributed the extreme prevalence rate, either too high or too low, to the following reasons: (1) poorly defined or inconsistent terms; (2) different measurement tools used to measure workplace bullying events; (3) under-reporting due to a lack of reporting system or fear of repercussions; (4) over-sensitive reporting. Therefore, researchers need to consider the study designs, socio-cultural, and organizational contexts when interpreting the prevalence rates. Additionally, it is good for researchers to consider measuring other indirect measures of workplace bullying, such as job satisfaction, intention to leave, etc.

### 4.2. Antecedents of Workplace Bullying among Nurses

Workplace bullying can stem from various triggering factors (antecedents) and develop through multiple sources. We identified at least five main types of antecedents. These five can be grouped under two main levels: individual and organizational antecedents (Table 3). Although Johnson (2011) and Samnani and Singh (2012) have suggested the role of societal-level antecedents, such as the societal culture of individualism versus collectivism [29,33], we concurred with the findings by Karatuna et al. that both individual and organizational antecedents exert an overlapping but greater immediate effect on workplace bullying than societal cultures or norms [16]. This proposition can also be explained by two dominant workplace bullying doctrines: the work environment hypothesis and the individual-dispositions hypothesis [31]. It is important to note that these antecedents were not mutually exclusive, but reflect the dynamic and mutual interactions between situational and individual factors within the workplace [31]. The findings from this summary review were also consistent with other rigorous reviews in other fields [2,16,30,34].

### 4.3. Consequences of Workplace Bullying among Nurses

This summary review also shows that workplace bullying has many detrimental consequences, not only in terms of the health and well-being of nurses, but also patient safety. For example, Lever et al. reported 45 studies highlighting the mental and physical problems that have afflicted nurses who encountered workplace bullying [8]. These issues could lead to more staff taking sick leave and providing less-than-effective care at work. In addition, Hutchinson and Jackson found 30 studies demonstrating how workplace bullying reduces nurses’ work performance and productivity and prevents effective teamwork and communication [17]. This inevitably creates a negative and hostile work environment, leading to organizational consequences, such as reduced job satisfaction, increased intention to quit, and staff turnover/attrition rate, which inevitably leads to higher organizational costs due to recruitment and retention difficulties [14,23].

### 4.4. Strengths and Limitations of This Umbrella Review

**Figure 2:**
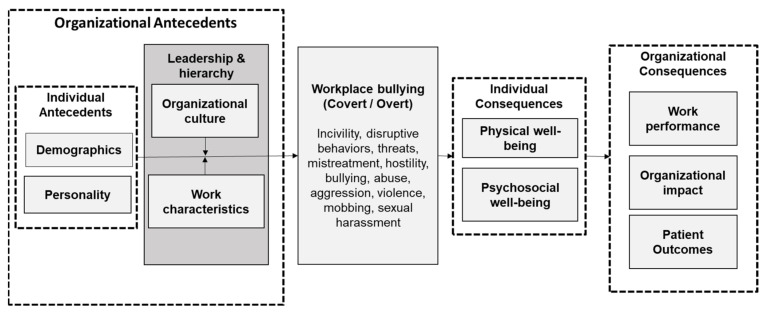
Conceptual Framework for Workplace Bullying among Nurses.

This is the first summary review to synthesize an extensive body of systematic reviews about workplace bullying to the best of our knowledge. We conducted a comprehensive search strategy and critical appraisal of the published reviews under the Cochrane Overview of Reviews method. Ultimately, we generated a conceptual framework to help clinicians and researchers understand the extent of research underlying this topic (Figure 2). However, this review is not without its limitations. First, we excluded several reviews that did not focus primarily on nurses, were published outside the last ten years, did not specify any systematic review methodology, or were published in non-English language [1,35,36,37]. We acknowledge that this could potentially result in the omission of several systematic reviews and their findings. Second, as we only included peer-reviewed journal publications, there is a possibility of publication bias, with studies reporting only positive results more likely to be published. These positive effects may be compounded in our included reviews [12]. Finally, we did not conduct a re-analysis of possible meta-analysis within the included reviews due to heterogeneity in measurement outcomes and study designs. This aspect may have limited the extent to which we could draw convincing conclusions about the review findings and any associations of variables within the conceptual framework.

### 4.5. Implications for Further Research

Bullying is a social phenomenon that has been extensively studied within nursing and non-nursing literature. This review found that current studies over-utilized cross-sectional survey designs and generated varied and conflicting results in the literature, making it difficult to determine whether the key correlates of bullying are predictors, consequences, or both. For example, there were times when the occurrence of bullying caused a poor work environment or times when it became vice versa [4,16]. Based on the review, the associations between bullying and correlates are likely characterized by reciprocal relationships. This finding aligns with bullying as a dynamic social phenomenon [2]. Therefore, there is a need for more advanced study designs where one can also identify and determine directionality between variables based on individual contexts.

Next, there is a need to design robust and effective interventions to address workplace bullying. Although this summary review did not extract systematic reviews focusing on workplace bullying interventions, we observed only a few reviews that addressed this issue. Additionally, these reviews only retrieved a few studies that reported bullying intervention’s effectiveness, highlighting a lack of studies in this area [38,39]. To achieve this, clinicians could consider using advanced and sound methodological designs and a well-developed theoretical framework [2]. Experimental research designs or survey studies following the same individuals over several time points (e.g., diary studies or longitudinal studies with multiple measurement points) are also needed to provide better indications of causality and intervention effectiveness [38,39].

## 5. Conclusions

This summary review evaluated the prevalence, antecedents, and consequences of workplace bullying among nurses based on an extensive body of systematic reviews published between 2013 and 2021. Workplace bullying was reported to affect at least one-quarter of the nursing population, higher than in other professions. The huge variation in prevalence rates from 1 to 90% reported across different reviews could be attributed to socio-cultural differences, workplace differences, heterogeneity in study designs, and operationalization of terms and measurement tools. The review findings on the antecedents and consequences demonstrated the complex and overlapping dynamics in the relationships among different variables for workplace bullying. We synthesized the findings from the included reviews and proposed an integrative model to explain this phenomenon and serve as the basis for future research.

## Figures and Tables

**Table 1:** Summary table for included systematic reviews.

S/N	Article	Objectives	Review Typology	Search Strategy	Number of Included Studies/Total Participants	Geographical Location	Findings
1.	Hutchinson & Jackson (2013) [17]	To examine the relationship between the various forms of hostile clinician behaviors and patient care.	A mixed-methods systematic review	8 databases (CINAHL, Health Collection (Informit), Medline (Ovid), Ovid, ProQuest Health and Medicine, PsycINFO, PubMed and Cochrane library), including hand searching of reference lists. Search period: Between 1990 and 2011. Inclusion: - Peer reviewed research. - English papers. - Unpublished masters or doctoral thesis. - Substantive reviews. - Addressed review questions. Exclusion: - Academic settings. - Did not address review question.	30 studies *N* = 102,909	USA (16), Australia (7), Canada (3), United Kingdom (1), New Zealand (1), Iceland (1), Finland (1)	Q3: Consequences on patient safety included: - Nurse engaging in avoidance behavior and delayed communication at work. - Erosion of patients’ confidence in nurses’ capability. - Nurses abusing their power over patients through yelling, swearing, or withholding privileges from vulnerable patients. - Other outcomes: reduced morale, intent to leave, productivity and caregiving error.
2.	Spector et al. (2014) [18]	To provide a quantitative review that estimates exposure rates by type of violence, setting, source, and world region.	A quantitative review	5 databases (Embase, MEDLINE, PsycINFO, PubMed and Web of Science). Search period: From earliest date to October 2012. Inclusion: - Primary studies to be concerned with violence in healthcare or nursing. - English papers. Exclusion: - Non-primary research. - Qualitative studies that did not include incidence rates.	136 studies *N* = 151,347	Worldwide	Q1: Prevalence & trends: Violence types are divided into: - Physical (95 studies). - Non-physical (81 studies). - Bullying and others (50 studies). - Sexual harassment (33 studies). - Injured (18 studies). Prevalence rates: - Physical violence (36.4%). - Non-physical (66.9%). - Bullying and others (39.7%). - Sexual harassment (25%). - Overall (50.5%). - Injured (32.7%). Geographical locations: - Highest rates for physical violence and sexual harassment in the Anglo region. - Highest rates of nonphysical violence and bullying in the Middle East. - Physical violence was most prevalent in emergency departments, geriatric, and psychiatric facilities.
3.	Trépanier et al. (2016) [4]	To provide an overview of the current state of knowledge on work environment antecedents of workplace bullying.	Systematic review with narrative synthesis	3 databases (PsycINFO, ProQuest and CINAHL). Search period: From earliest date to 2014. Inclusion: - Focuses on WB. - Empirical studies to be concerned with review questions. - English papers. Exclusion: - Exclude terms such as incivility and violence.	12 studies *N* = 4177	North America (7), Australia (3), Turkey (2)	Q2: Identified four main categories of work-related antecedents of workplace bullying: (a) job characteristics, (b) quality of interpersonal relationships, (c) leadership styles, and (d) organizational culture.
4.	Houck & Colbert (2017) [3]	To explore and synthesize the published articles that address the impact of workplace nurse bullying on patient safety.	Integrative review	5 databases (PubMed, CINAHL, PsycINFO, Cochrane library and MEDLINE). Search period: Between 1995 and March 2016. Inclusion: - Original research in the last 20 years. - English papers.	11 studies *N* = 16,137	USA (7), Australia (2), Canada (1), and United Kingdom (1)	Q3: The effect of bullying on nurses’ work was not sufficient to reveal all risks to patient safety. - Error in treatment or medication (6 studies). - Silences or inhibits communication (5 studies). - Adverse event/patient mortality (4 studies). - Patient satisfaction/complaints (2 studies). - Altered thinking/concentration (2 studies). - Delayed care (1 study). - Patient falls (1 study).
5.	Pfeifer & Vessey (2017) [19]	To synthesize and evaluate the existing literature on workplace bullying and lateral violence in the Magnet^®^ setting.	Integrative review	5 databases (CINAHL, MEDLINE, PsycINFO, Cochrane library and Web of Science). Search period: Between January 2008 and February 2017. Inclusion: - English papers only. - Research studies. Exclusion: - Studies that did not directly examine both Magnet organizations and the concepts of WB.	11 studies *N* = 7657	USA	Q2: Magnet nurses reported lower WB scores than nurses working in non-Magnet organizations (based on four studies).
6.	Bambi et al. (2018) [20]	To detect specifically the prevalence of workplace incivility (WI), lateral violence (LV), and bullying among nurses.	Narrative review	3 databases (MEDLINE, CINAHL and Embase). Search period: No time limitation. Inclusion: - Italian and English papers. - Quantitative studies, original mixed-methods studies, systematic reviews, and meta-analysis. Exclusion: - Nursing students. - Academic settings. - Qualitative studies. - Secondary literature.	Workplace incivility:16 studies *N* = 12,246 Lateral violence:25 studies *N* = 25,375	Workplace incivility: Canada (8), USA (5), China (1), Egypt (1), Pakistan (1) Lateral violence: USA (15), Europe (5), Asia (2)—Turkey & South Korea, South Africa (1), New Zealand (1), Jamaica (1)	Q1: Prevalence: - Lateral violence has a prevalence ranging from 1 to 87.4%. - Bullying prevalence varies between 2.4% and 81%. Q3: Consequences: - 10% of bullied nurses develop post-traumatic stress disorder symptoms (psychosocial well-being). - WB is positively correlated with burnout (β = 0.37 *p* < 0.001). - WB reduces job efficiency (r = −0 322, *p* < 0.01). - 78.5% of bullied nurses with < 5 years resigned. - Bullied nurses were 1.5 times more likely to report absenteeism.
7.	Hartin et al. (2018) [14]	To discuss the current state of knowledge about bullying in the nursing profession in Australia.	Integrative review	3 databases (MEDLINE, CINAHL and Scopus). Search period: Between January 1991 and December 2016. Inclusion: - English papers. - Primary research. - Addressed research topics. - Studies conducted in Australia. Exclusion: - Studies published after 2016. - Non-English papers.	23 studies *N* = 16,168	Australia	Q1: 61% of respondents reported WB within the last 12 months. Nurse-to-nurse aggression was the most distressing type of bullying, and statistics were likely to be under-reported. Q3: - Individual impact: Consists of 4 dimensions (psychological, physical, emotional, and social). WB increased prevalence of psychological distress, depression, and burnout. It reduced motivation, self-worth, and work ethic. - Work: Decreases job satisfaction, motivation, and work productivity. Among them, 24% considered resigning over the next four weeks. - Organizational-level: Hostile workplace increased absenteeism, and decreased productivity, in addition to recruitment and retention difficulties.
8.	Crawford et al. (2019) [21]	To examine the evidence regarding nurse-to-nurse incivility, bullying, and workplace violence for the 4 nursing populations (student nurses, new graduate nurses, experienced nurses, and academic faculty).	Integrative review	6 databases (CINAHL, Cochrane library, Embase, ERIC, PsycINFO, and PubMed). Include Google Search. Search period: Between 2010 and 2016. Inclusion: - English papers. Exclusion: - Studies that did not answer the clinical question. - Studies that did not focus on the concepts of incivility, hostility, and/or workplace violence. - Studies conducted outside the acute care environment. - Studies based outside USA or Canada. - Studies that included healthcare professionals other than nurses.	21 studies *N* = Not reported	USA and Canada	Q1: No number reported. Highlighted that WB prevalence rates among nurses have not changed in more than 20 years. Q2: Antecedents were divided into 3 layers of WB triggers: organizational, work environment, and personal. Suggested that young nurses were at a higher risk. Q3: Identified 84 negative academic, organizational, work unit, and personal outcomes. Others: - Lack of unifying definition of the terms surrounding WB. - Leadership plays a mediating role in the WB triggers and environment.
9.	Hawkins et al. (2019) [22]	To synthesize evidence on negative workplace behavior experienced by new graduate nurses in acute care setting and discuss implications for the nursing profession.	Integrative review	5 databases (CINAHL, MEDLINE, ProQuest, JBI and Scopus). Search period: Between 2007 and 2017. Inclusion: - Original research. - Involved new graduate nurses of <2 years of experience. Exclusion: - Non-research papers. - Non-English papers.	16 studies (14 published articles & 2 dissertations) *N* = 3043	Canada (6), USA (3), Australia (2), Taiwan (2), Ireland (1), South Korea (1), Singapore (1)	Q1: - Prevalence ranged widely from 0.3 to 73.1%. These variations depend on the context and instrument measuring WB occurrences (e.g., daily basis, over the past 1 month or 12 months). For those studies measuring WB within past 6–12 months, prevalence ranged between 25.6% and 73.1%. - Manifestation of WB divided into personal or professional attack (highlighted in Table 3). Q2: Three antecedents were identified: - New graduates’ perceived lack of capability. - Magnifying power and hierarchy. - Leadership style and influence of management. Q3: Individual impact and patient care identified: - Emotion/psychological outcomes (low self-esteem, anxiety, distress, depression, disempowerment). - Workplace (job satisfaction, burnout, turnover intentions, absenteeism, attrition). - Patient care (distraction, poor work concentration, willingness to seek help and engage in work). Others: - Theoretical frameworks used in WB literature included: (a) social capital theory; (b) incivility spiral; (c) authentic leadership; (d) oppression theory; (e) Six Areas of Worklife model. - Conceptual differences and variety of the terms within literature.
10.	Lever et al. (2019) [8]	To review both mental and physical health consequences of bullying for healthcare employees.	Systematic review (quantitative studies)	5 databases (Embase MEDLINE, PsycINFO, PubMed and Web of Science). Search period: Between 2005 and January 2017. Inclusion: - Primary studies published in peer-reviewed journals. - Studies that addressed the review questions. Exclusion: - Non-English papers. - If studies were inaccessible in full text. - Not conducted in healthcare settings.	45 studies *N* = varied from 61 to 9949.	15 studies in North America (Canada-10; USA-5) 15 studies in Europe (UK-4; Denmark-4; Norway-3; Portugal-2; Germany-1; Bosnia-1) 6 studies in Australia (6) 7 studies in Asia (Turkey-4; Japan-2; China-1) 2 studies (Mixed profiles)	Q1: Prevalence: - Bullying prevalence ranged from 3.9 to 86.5%, with a pooled mean estimate of 26.3%. - Pooled prevalence for WB among nurses is 30.8% across 14 studies. - Pooled mean prevalence of WB in Asia was 47.1%, Australia 36.1%, Europe 18.4%, and North America 24.5%. Q3: Consequences divided into 2 types: - Mental health (anxiety, psychological distress, burnout, depression, suicidal ideation/attempts). - Physical health (sleep disorders, headache, gastrointestinal problems). - Bullied staff are more likely to take sick leave (because of mental or physical health disorders).
11.	Johnson & Benham-Hutchins (2020) [23]	To examine the influence of nurse bullying on nursing practice errors and patient outcomes.	A systematic review (involving qualitative synthesis)	4 databases (CINAHL, MEDLINE, Cochrane Library, and PsycINFO). Search period: Between January 2012 and November 2017. Inclusion: - Studies examining bullying among healthcare professionals, nurses as study participants. - Conducted in clinic or hospital settings. Exclusion: - Studies with students or clinicians other than nurses (e.g., physicians).	14 studies *N* = Not reported	Not reported	Q1: ED setting: 60% of respondents reported WB events. OR setting: 59% witnessed WB events, but only 6% self-reported such events in perioperative environment in the USA. Two types of bullying trends were identified: (a) work-related bullying originating from workplace environment, and (b) person-related bullying originating from informal personal relationships. Q3: Consequences included: - Individual: Psychosocial consequences of bullying include symptoms such as increased stress, somatic symptoms, frustration, absenteeism, and lack of concentration. - Workplace: WB has a strong inverse relationship with perceived peer relations - Patient outcomes: WB has a direct relationship with perceived errors and adverse events.
12.	Karatuna et al. (2020) [16]	To examine WB research among nurses with the focus on sources, antecedents, outcomes, and coping responses from a cross-cultural perspective during the years 2001–2019.	A cross-cultural scoping review	4 databases (CINAHL, PubMed, PsycINFO and Web of Science). Search period: Between 2001 and 2019. Inclusion: - Primary studies published in peer-reviewed journals. - WB defined within the concept of violence and aggression. Exclusion: - Studies that did not present empirical data. - Did not address the research questions. - Were not solely conducted among nurses. - If they were conducted among nursing students, faculty, or school nurses. - Did not have abstract and/or inaccessible in full text.	166 studies *N* = Not reported	29 countries worldwide, although research was mostly conducted in the Anglo cluster	Q2: Antecedents varied across cultures and classified as: (a) individual (demographics and personality traits); (b) organizational (leadership, work characteristics, and organizational culture). Other results included: - WB has an inverse relationship with nurses’ length of service and age. - Vertical bullying was most prevalent in higher power distance cultures (Eastern Europe and Southern Asia). - Horizontal bullying was either more or equally prevalent in lower power distance cultures (Confucian Asia). - Individual antecedents were more frequently reported in high collectivist cultures. - Organizational antecedents were similar across all cultures and highly dependent on workplace culture and environment. - Anglo countries tended to address WB events as they were seen as highly performance-oriented cultures. Q3: Consequences: - Negative outcomes of WB were very similar across different cultures and classified as follows: (a) work-related outcomes; (b) health and well-being related outcomes.

**Legend:** WB (Workplace bullying); USA (United States of America); WI (workplace incivility), LV (lateral violence); ASSIA (Applied Social Sciences Index and Abstracts); BSP (Business Source Premier); CINAHL (Cumulated Index to Nursing and Allied Health Literature); Embase (Excerpta Medica database); JBI (Joanna Briggs Institute); MEDLINE (Medical Literature Analysis and Retrieval System Online); IBSS (International Bibliography of the Social Sciences); Q1 (Question 1—What are the prevalence in workplace bullying in nursing studies?) Q2 (Question 2—What are the antecedents for workplace bullying in nursing?); Q3 (Question 3—What are the consequences of workplace bullying in nursing?).

**Table 2:** Quality appraisal of included systematic reviews.

S/N	Article	Quality of Study Using ROBIS Tool					Strengths	Limitations
		D1	D2	D3	D4	O		
1.	Hutchinson & Jackson (2013) [17] Mixed-methods systematic review	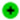	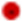	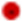	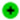	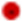	- A review that focused on how WB events could impact patient safety indirectly by affecting nurses’ behavior and performance. - Involved 30 studies with high sample size of 102,909. - Documented various forms of affected nurses’ performance that could compromise patient safety outcomes as a result of WB, e.g., avoidance or delayed communication at work.	- Mostly narrative synthesis. - Difficult to examine hostile behaviors and reliable patient safety outcomes due to variability in conceptualization and measurement of data. - More robustly designed studies are needed to conclude relationships between factors, nurses’ work environment, and patient care.
2.	Spector et al. (2014) [18] Quantitative review	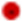	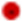	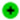	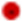	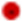	- Comprehensive review in establishing WB prevalence (nurse exposure to WB events) involving 136 studies and a sample size of 151,347 nurses. - Reported regional and country differences in the incidence rates and sources of violence, and suggested the role of sociocultural influences for WB events.	- Heterogeneity observed in study designs and quality. - Studies were not all comparable across type, setting, source, and region. - Little standardization in conceptualization, measures, methods across studies. - Did not find any study that specifically examined hostile behaviors and reliable secondary sources of outcome data.
3.	Trépanier et al. (2016) [4] Systematic review with narrative synthesis	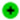	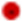	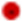	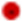	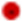	- A review that focused on how workplace environment antecedents could influence WB events. - Proposed a useful integrative model of workplace bullying in nurses. - Highlighted four main categories of work-related antecedents of workplace bullying: job characteristics, quality of interpersonal relationships, leadership styles, and organizational culture.	- Mostly narrative synthesis. - Confined search to three databases only, limiting its comprehensiveness in study findings. - Limited studies (*n* = 12), which were insufficient to justify the model development.
4.	Houck & Colbert (2017) [3] Integrative review	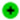	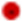	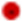	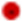	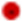	- A review that focused on how WB events could impact patient safety indirectly by affecting nurses’ behavior and performance. - Documented evidence to affirm the presence of WB in the hospital environment.	- Patient safety measures were primarily reported as staff-perceived outcomes and seldom related to direct patient measures. - Confined to Anglo countries. - Insufficient studies to support findings (*n* = 11). - Review was also constrained by inconsistent definitions and methodologies.
5.	Pfeifer & Vessey (2017) [19] Integrative review	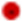	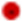	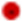	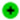	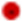	- Focus on role of healthy workplace (Magnet workplace) in mitigating WB events. - Proposed an emerging concept, accountability, as a possible area for WB interventions. - Results were well-synthesized according to the review objectives.	- Studies confined to a single country (USA), limiting its generalizability. - Authors highlighted that existing studies were limited (only 11 studies) and lacked methodological rigor to conclude the findings. - More studies are needed to establish relationship between workplace and WB events.
6.	Bambi et al. (2018) [20] Narrative review	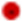	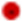	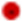	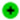	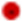	- A review that focused on WB prevalence for incivility and violence among nurses with 16 and 25 studies, respectively. - Review also detailed classification of 3 main consequences of WB for individual nurses (physical, psychological, and behavioral). - Sample size was large and sufficient to validate findings within North American context.	- Limited to Canada and the USA context, with limited generalizability to other regions. - Confined search to three databases only, limiting its comprehensiveness in study findings. - Did not grade level of evidence for studies.
7.	Hartin et al. (2018) [14] Integrative review	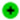	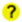	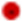	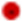	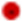	- Review examined WB among nurses in Australia with 23 included studies.	- Mostly narrative synthesis. - Confined search to three databases only, limiting its comprehensiveness in study findings. - Limited to Australian workplace context. - A lack of a clear definition prevents a full understanding of this construct.
8.	Crawford et al. (2019) [21] Integrative review	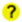	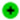	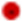	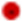	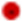	- Specific review that examined the evidence regarding WB events for four nurse populations: student, new graduate, experienced, and academic faculty. - Mostly narrative synthesis of findings from 21 studies. - Provided a logical and clear classification for WB antecedents and outcomes for readers.	- Evidence was graded using validated tool, but not specified in Table 1 within the article, but there might be a potential risk of bias on lack of clarity over data collection and quality appraisal process. - Conclusion and recommendations for practice were not clearly articulated, with the exception of the role of nursing leadership.
9.	Hawkins et al. (2019) [22] Integrative review	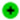	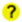	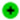	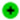	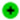	- Specific review focused on WB prevalence among new graduate nurses in acute care settings. - Rigorous review process, and utilized validated tool for quality appraisal. - Findings were comprehensive and addressed most review questions.	- Relevant ‘grey’ literature was not included. - Limited studies of different methodology only (*n* = 16). - Low sample size of 3043. - Lack of studies on interventions to address WB.
10.	Lever et al. (2019) [8] Systematic review (quantitative studies)	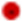	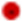	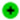	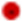	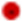	- Specific review focused on mental and physical consequences of bullying for healthcare staff. - Sufficient sample of 45 included studies with a large, pooled sample size. - Included five databases.	- Not specific to nurses. - WB definition not standardized across all included papers. - Most papers were cross-sectional in nature.
11.	Johnson & Benham-Hutchins (2020) [23] Systematic review (involving qualitative synthesis	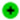	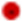	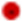	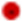	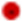	- Specific review focused on WB impact on nursing errors. - Utilized quality appraisal tool, but did not provide information about it. - Generated findings specific to emergency department and operating room settings, which were viewed as highly stressful areas and not well researched.	- Included only four databases. - Included studies were limited due to specific inclusion and exclusion criteria (*n* = 14). - Utilized quality appraisal tool, but did not provide information about it. - Did not include grey literature. - Did not include non-English papers.
12.	Karatuna et al. (2020) [16] Scoping review	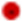	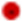	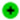	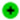	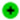	- Comprehensive search involving 166 studies across the world. - Examined the social determinants for WB trends and prevalence in different regions. - Well-synthesized findings.	- Included only four databases. - Included only English papers. - Did not include grey sources, or unavailable literature.

**Legend:** D1 (Domain 1: Study eligibility criteria); D2 (Domain 2: Identification and selection of studies); D3 (Domain 3: Data collection and study appraisal); D4. (Domain 4: Synthesis and findings)’ O (Overall: Risk of bias in the review) 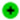: Low risk for bias; 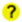: Some concerns for bias; 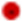: high risk for bias.

**Table 6:** Summary of concepts, terms, measurement tools, and theories for workplace bullying in nursing and non-healthcare literature.

Concepts/Terms	Examples
Sources	Management, leaders, peers, non-nursing colleagues, patients, and family members
Direction	Horizontal, lateral, and vertical
Manifestations	Incivility, disruptive behaviors, threats, mistreatment, hostility, bullying, abuse, aggression, violence, mobbing, sexual harassment
Forms	Covert behaviors (e.g., sabotage, withholding support) and overt forms (verbal and physical)
Measurement instruments	Secondary data extracted from incident-reporting system or formal reports Self-labeling method—single-item of “yes/no” or “frequency” Negative Acts Questionnaire * The Bullying Inventory for the Nursing Workplace * Leymann Inventory of Psychological Terror Workplace Harassment Scale
Theories	Blau’s (1964) Social Exchange Theory Seligman and Maier’s (1967) Theory of Learned Helplessness Freire’s (1970) Oppression Theory * Karasek’s (1979) Job Demand–Control Model Cohen and Felson’s (1979) Routine Activities Theory Lazarus and Folkman’s (1984) Transactional Stress Theory * Glasl’s (1982) Nine-Stage Model Of Conflict Escalation Leymann’s (1996) Work–Environment Hypothesis Weiss and Cropanzano’s (1996) Affective Event Theory Hobfoll’s (2001) Conservation of Resources Theory Lutgen-Sandvik’s (2003) Employee Emotional Abuse (EEA) Model (extension of Leymann’s Model) De Dreu, van Dierendonck, and Dijkstra’s (2004) Dual Concern Theory Leiter and Maslach’s (2004) original Six Areas of Worklife Model * Ursin and Eriksen’s (2004) Cognitive Activation Theory of Stress Bakker and Demerouti’s (2007) Job Demand Resource Model Kerber et al. (2015) Social Capital Theory * Ryan and Deci’s (2019) Self- Determination Theory

* more commonly used in nursing literature.
